# P21 My eye was cloudy at first and I can see it is melting away!

**DOI:** 10.1093/rap/rkab068.020

**Published:** 2021-10-19

**Authors:** Phing Sue Wong, Voon Ong, Richard Stratton

**Affiliations:** Royal Free Hospital, London, United Kingdom

## Abstract

**Case report - Introduction:**

Eye involvement represents a common finding in patients with systemic autoimmune diseases, particularly rheumatoid arthritis, Sjogren syndrome, seronegative spondyloarthropathy, and variable vasculitides e.g., sarcoid, antineutrophil cytoplasmic antibody (ANCA)-associated vasculitis, GCA, Behcet’s and Cogan’s syndrome.

Eye diseases can precede the clinical manifestation of systemic disease or vice versa; some remain as a single organ entity with serological positivity.

Accurate diagnosis and timely treatment is crucial in visual threatening conditions including corneal melting, which is also named peripheral ulcerative keratitis (PUK). Others include uveitis, scleritis, chorioretinitis and optic neuritis.

**Case report - Case description:**

Miss KA, a 57-year-old female patient was seen in our clinic in 2009 with symmetrical inflammatory polyarthritis of more than 5 months duration. She took celecoxib and tramadol for her symptoms. She also has knee osteoarthritis and fibromyalgia with multiple tender points.

Clinical examination reviewed synovitis over bilateral metacarpophalangeal, proximal interphalangeal and distal interphalangeal joints.

Hand imaging reviewed no erosion, chest X-rays was normal.

ESR 37, CRP 10. ACPA was positive.

She immediately was started on methotrexate with later add-on hydroxychloroquine.

A year into treatment, her DAS28 score remain high at 5.9-6.3 and still depended on intermittent rescue systemic steroid. We decided to escalate her treatment to bDMARDs. The oatient received INH prophylaxis for positive TB ELISPOT.

She was on etanercept for 5 years and later switched to rituximab as the patient developed eosinophilic dermatosis.

8 years down the road in 2017, the patient has acute left eye redness and was diagnosed corneal melting by ophthalmologist. She received prednisolone 60mg daily, methotrexate and adalimumab. Unfortunately, 2-years later her right eye was affected. Her ESR was 53; however, clinically and ultrasound so no active synovitis.

She received surgery treatment for right eye PUK. She was also later given parenteral cyclophosphamide 6 cycles for progression of PUK.

After that, her keratitis is stable but unfortunately synovitis has relapsed.

She currently remains on methotrexate, systemic steroid.

We decided to apply funding for L6 inhibitor.

**Case report - Discussion:**

Systemic autoimmune diseases affect approximately 7—9% of the UK population. It has strong female predominance and has a broad spectrum of symptom manifestations including ocular disease. Eye involvement can be the first manifestation due to inflammatory, vascular or the consequence of infectious process.

Certain ocular diseases allow prediction of underlying systemic diseases due to their prevalence. Examples are anterior uveitis in seronegative spondyloarthritis and sarcoidosis, episcleritis for rheumatoid arthritis and hypopyon in Behcet's disease. Sometimes, ocular inflammation can be its own monogenic autoinflammatory diseases.

The commonest eye disease in RA is secondary Sjogren, episcleritis and scleritis. Rarer conditions are uveitis and keratitis.

Keratitis in RA is often the consequence of underlying scleritis. Its complications range from corneal opacification to thinning, and it leads to the most severe form of necrotizing keratitis called peripheral ulcerative keratitis (PUK), corneal melting. Patients often complain of red eye, ocular pain, tearing, blurred or decreased vision, or photophobia resembling scleritis.

It is a sight threatening condition and should immediately be treated with high-dose systemic steroid.

There is no consensus for treating PUK associated with RA. Systemic corticosteroids at a dose of 0.5—1mg.kg are routinely prescribed. Various immunosuppressive agents reported in literature are cyclophosphamide, methotrexate, azathioprine, mycophenolate mofetil, and calcineurin inhibitors, like cyclosporine and tacrolimus. Biologics used are a group of anti-TNF inhibitors: infliximab, adalimumab, golimumab and certolizumab pegol, and have also been reported to be effective.

Adjunct therapies include topical therapies with corticosteroid, cyclosporine and tacrolimus with surgical procedures. It is required in cases of impending corneal perforation to preserve globe hence the patient’s visual integrity. Surgical procedures are also for repair of sequalae of perforation. It is often undertaken post active inflammation.

**Case report - Key learning points:**

1. Ocular manifestation is common in systemic autoimmune diseases and not foreign to rheumatologists. It can present before clinical manifestation of systemic disease, or along the clinical course. Some manifest as monogenic ocular disease.

2. Often, rheumatologists co-manage these cases with an ophthalmologist, mainly looking for underlying disease of rheumatological conditions. In addition to that, rheumatologists are more experienced in managing patients on systemic immunosuppressant including biologics.

3. Accurate diagnosis of visual threatening ocular inflammation by an experienced ophthalmologist treating eye diseases related to rheumatological conditions is crucial. This will allow on-time institution of anti-inflammatory drugs using a topical systemic steroid with or without other immunosuppressants.

4. There is no consensus in treating PUK; commonly used immunosuppressants and anti-TNF have been reported to be successful in cases. Etanercept however is to be used cautiously as it can worsen or induce new onset of uveitis.

5. The visual and life prognosis in patients with PUK and another inflammatory ocular issues are improving in the biologic era.

3. Surgical procedure can be an initial emergency measure in the case of severe sight-threatening situations or reparative measures after active inflammatory phase of disease. Measures include the use of a tissue adhesive bandage contact lens, corneal graft and corneal transplant.

